# The Impending Obsolescence of Traditional Dentistry: A Forecast of Artificial Intelligence Integration and Its Transformative Impacts

**DOI:** 10.7759/cureus.100547

**Published:** 2026-01-01

**Authors:** Aleksandar Naydenov, Todor Uzunov, Ilia Liondev

**Affiliations:** 1 Department of Prosthetic Dental Medicine, Medical University - Sofia, Sofia, BGR

**Keywords:** artificial intelligence, automation, dentistry, forecast, job displacement

## Abstract

The integration of artificial intelligence (AI) into dentistry is revolutionizing oral healthcare and is evolving from narrow, task-specific models to potentially artificial general intelligence (AGI)-led practices. We hypothetically outline four stages describing the possible evolution of AI in dentistry: (1) competing in diagnostics (2025-2035); (2) competing in virtual planning and construction (2025-2040); (3) competing in real work execution (2025-2040); and (4) AGI may take the lead in some task-specific dental procedures (2025-2047).

## Editorial

The integration of artificial intelligence (AI) into dentistry is reshaping oral healthcare, introducing significant advancements in diagnostics, treatment planning, procedural execution, and dental practice management [1]. These systems are used to detect dental caries and periapical lesions from X-rays, predict orthodontic outcomes, design prosthetic restorations, and automate administrative processes such as scheduling and patient communication [2]. Some researchers are exploring even more advanced AI systems, including multimodal AI that integrates diverse data sources [3] and robotics for autonomous procedures, with the long-term potential of artificial general intelligence (AGI) to perform all intellectual tasks a human dentist can undertake [4]. We hypothetically outline four stages describing the possible evolution of AI in dentistry. Each stage represents a conceptual scenario based on observed trends, not a prediction.

**Figure 1:**
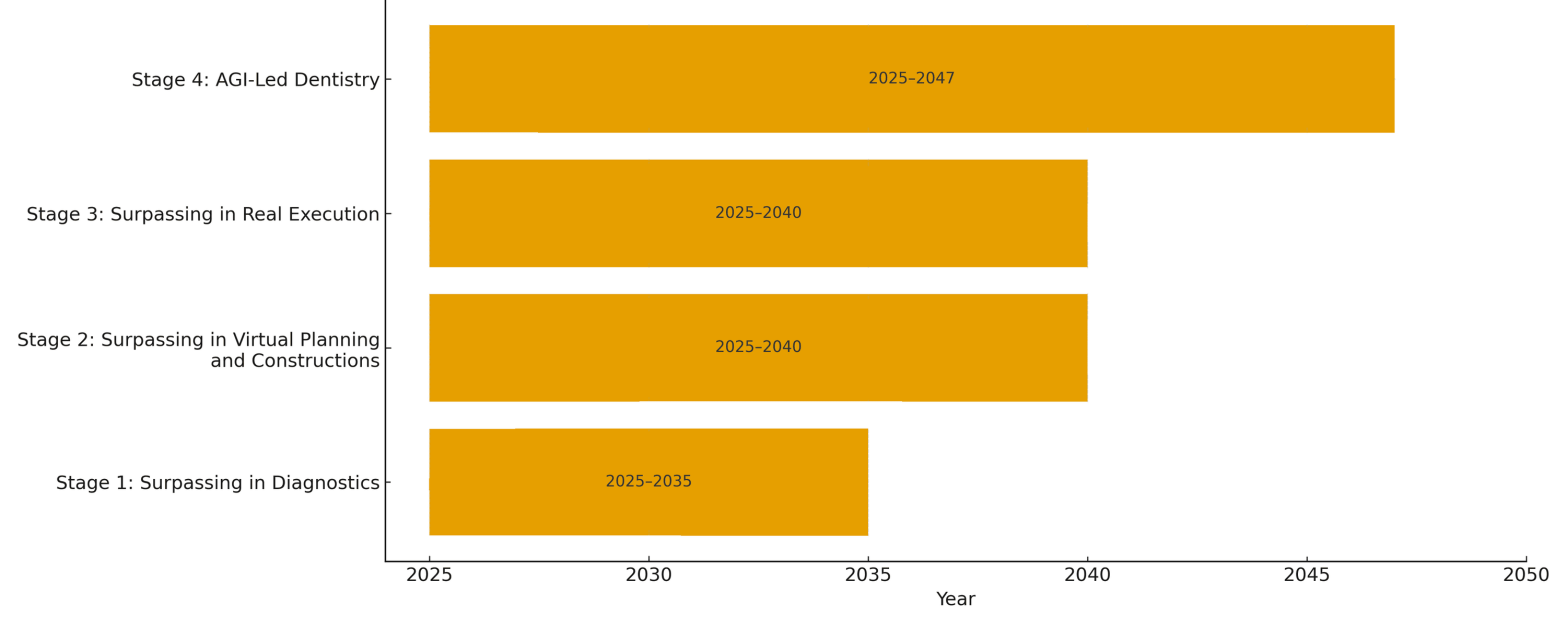
Timeline of the four stages representing AI evolution in dental medicine AGI, artificial general intelligence; AI, artificial intelligence

Our forecasts of how AI may surpass human capabilities in dentistry are based on current trends in machine learning and deep learning advancements, robotics, and data integration. The timeline of AI evolution stages is shown in Figure 1.

Stage 1: Competing in diagnostics (2025-2035)

AI is already competing with dentists in specific diagnostic tasks, such as predicting tooth extractions from X-rays [5] and detecting periapical lesions [6]. AI also competes with human specialists in specific diagnostic tasks for oral cancer, particularly in analyzing histopathological images and CT scans [7]. Around 2028, AI systems such as ChatGPT may equal skilled humans in scientific writing and predictive algorithms, with broader competition in health data integration by 2030-2035 [8].

Stage 2: Competing in virtual planning and construction (2025-2040)

AI tools may compete with dentists and dental technicians in designing prosthetic constructions. Autonomy of the workflow in limited, task-specific domains can be expected around 2035-2040. AI may achieve expert-quality implant planning with greater time efficiency and consistency than humans [9]. AI-generated smile designs have been found to be comparable to manually created designs [10]. A Global Trends report suggests that these AI-augmented processes may compete with traditional human-led design in speed, accuracy, and personalization for medical tools such as implants [11].

Stage 3: Competing in real work execution (2025-2040)

Even today, AI-assisted robots may rival traditional static-guided implant surgery in precision [12]. In endodontics, AI assists root canal therapy; in orthodontics, AI is used for bending archwires in malocclusion treatment; in oral radiology, AI is used to assist in positioning X-ray equipment and performing subtraction radiography [13]. The Imagining the Digital Future Center at Elon University wrote, “By 2040, almost 75% of all employees will be laid off and replaced with AI. There will be hospitals with virtually no doctors, only nurses and AI” [14].

Stage 4: AGI may take the lead in some task-specific dental procedures (2025-2047)

AGI is characterized by its flexibility, autonomy, and ability to handle unfamiliar tasks without requiring task-specific programming [15]. A survey of 2,778 AI researchers reported a median expectation of AGI becoming real by 2047, with a 10% chance by 2027 [16]. Some industry leaders expect an even earlier emergence of AGI. OpenAI’s Sam Altman stated in 2025 that AGI could arrive by 2025-2030 [17], while Google DeepMind’s Demis Hassabis estimates AGI development within five to 10 years (2030-2035) [18]. AGI’s ability to perform certain limited, task-specific dental procedures may reduce demand for human involvement in some procedures, such as caries detection and implant planning. The integration of AI in dentistry introduces the possibility of job displacement, which is considered one of the main disadvantages, as well as new challenges in doctor-patient relationships [19].

Evidence cited within each stage comes from validated studies, but extrapolations regarding timing, scope, and workforce impact are speculative. The timelines are hypothetical and not modeled and are intended to stimulate discussion about possible futures rather than assert inevitability or precise prediction.

Nevertheless, beyond dentistry, AI is beginning to replace humans in executing specific tasks across various professions, marking a significant shift. This development raises questions about the future roles of dentists and dental technicians as AI competes with human capabilities in tasks traditionally considered exclusive to human intellect [20]. It must be noted that the four hypothetical stages presented have important limitations. Some referenced viewpoints, particularly those from industry leaders, reflect non-scientific opinions that may introduce bias. Additionally, the number of high-impact studies in several areas of AI-driven dentistry remains limited.

In conclusion, this article provides a broad overview of AI’s potential transformation of dentistry through a structured, hypothetical four-stage framework. While it demonstrates thoughtful consideration of future developments, its scientific credibility could be enhanced through stronger empirical evidence. Future scientific publications on this topic would improve its robustness as a scientific contribution.
